# Proteomic profiling of brain organoids and extracellular vesicles identifies early Alzheimer's disease biomarkers and drug response heterogeneity

**DOI:** 10.1002/alz.71273

**Published:** 2026-04-08

**Authors:** Rachel J. Boyd, Daiyun Dong, Ram Sagar, Anton Iliuk, Waqar Ahmed, Xenia Androni, Anton P. Porsteinsson, Paul B. Rosenberg, Constantine G. Lyketsos, Kenneth W. Witwer, Vasiliki Mahairaki

**Affiliations:** ^1^ Department of Genetic Medicine Johns Hopkins University School of Medicine Baltimore Maryland USA; ^2^ The Richman Family Precision Medicine Center of Excellence in Alzheimer's Disease Johns Hopkins University School of Medicine Baltimore Maryland USA; ^3^ Division of Geriatric Medicine and Gerontology, Johns Hopkins School of Medicine Baltimore Maryland USA; ^4^ Tymora Analytical Operations West Lafayette Indiana USA; ^5^ University of Rochester School of Medicine and Dentistry Rochester New York USA; ^6^ Department of Psychiatry and Behavioral Sciences Johns Hopkins University School of Medicine and Johns Hopkins Bayview Medical Center Baltimore Maryland USA; ^7^ Department of Molecular and Comparative Pathobiology Johns Hopkins University School of Medicine Baltimore Maryland USA; ^8^ Department of Neurology Johns Hopkins University School of Medicine Baltimore Maryland USA

**Keywords:** Alzheimer disease, clinical heterogeneity, escitalopram oxalate, extracellular vesicles, extracellular vesicle proteomics, induced pluripotent stem cells, proteomic profiling, serotonergic hindbrain organoids

## Abstract

**INTRODUCTION:**

Alzheimer's disease (AD) exhibits high genetic and clinical heterogeneity that limits therapeutic success. Patient‐derived brain organoids and their extracellular vesicles (EVs) provide physiologically relevant models to study disease mechanisms and individualized drug responses.

**METHODS:**

We generated the largest brain organoid cohort to date, derived from 30 independent induced pluripotent stem cell (iPSC) lines from AD and control individuals. Comparative proteomic profiling was performed on both organoids and their secreted EVs to capture molecular diversity and treatment effects.

**RESULTS:**

Organoids and EVs consistently recapitulated neuronal proteomic signatures and revealed early alterations in AD‐related pathways, including synaptic and neurotransmitter dysfunction. Distinct proteomic responses mirrored individual variability in selective serotonin reuptake inhibitor sensitivity.

**DISCUSSION:**

Integrating organoid and EV data provides a systems‐level view of AD pathophysiology and treatment response, positioning this dual‐platform model as a cost‐effective tool for precision medicine and drug discovery.

## Highlights

- We developed the largest brain organoid and extracellular vesicle cohort to date, from 30 donors.
- This is the first study to generate analogous proteomic data from organoids and their extracellular vesicles (EVs).
- Patient‐derived serotonergic hindbrain organoids model Alzheimer's disease (AD) heterogeneity.
- This organoid–EV platform detects early disease pathways and individual drug responses.
- This model enables scalable, precision drug screening and biomarker discovery for AD.

## 1 BACKGROUND

The complex genetic basis of Alzheimer's disease (AD) continues to impede the development of broadly effective therapies against what is the most common neurodegenerative disease in humans. More than 90 genomic loci have been implicated in AD risk,^1^, ^2^ which presents in mid‐to‐late adulthood and encompasses a variable range of clinical symptoms. For example, a subset of AD patients manifest neuropsychiatric symptoms (NPSs), including anxiety, depression, and agitation, while others do not.^3^

NPSs in AD are associated with accelerated disease progression and worse outcomes for patients and caregivers.^4^ Pathological studies have demonstrated that damage to monoamine/serotonin‐producing dorsal raphe nuclei occurs early in AD, and that patients with NPSs exhibit dysfunctional serotonergic systems that contribute to symptom emergence.^5^, ^6^, ^7^ Among AD patients, variability in response to selective serotonin reuptake inhibitors (SSRIs) highlights the need for more individualized approaches.^8^, ^9^, ^10^ Identifying disease subtypes that are therapeutically relevant and mechanistically informative, through biomarkers that predict NPSs or response to SSRIs, could markedly improve patient outcomes.^11^ However, the considerable clinical heterogeneity observed in AD reflects its complex and multifactorial genetic architecture, underscoring the limited construct validity of traditional preclinical animal models that are largely based on highly penetrant, monogenic variants.

Recent advances in stem cell technologies offer a transformative platform for advancing precision medicine in AD. Induced pluripotent stem cells (iPSCs) derived from patient blood samples can be differentiated and grown into 3D brain organoids, providing genetically relevant models to study variability in disease mechanisms and therapeutic responses.^12^, ^13^, ^14^ Unlike traditional two‐dimensional neuronal cultures, brain organoids more faithfully recapitulate the cellular architecture and complex cell–cell interactions of the human brain, making them a more physiologically relevant system for modeling variability in neurodegenerative disease.^15^, ^16^, ^17^ Furthermore, brain organoids can be generated by applying guided differentiation protocols that use patterning factors and signaling molecules to direct iPSCs toward region‐specific neural identities, including the forebrain,^13^ midbrain,^18^ hindbrain,^9^ or cortex.^19^ Patient‐derived brain organoids both recapitulate many disease‐specific phenotypes^20^, ^21^, ^22^ and offer insights into biomarkers that distinguish patients from unaffected individuals or defined AD subgroups, potentially revolutionizing early disease detection strategies.

Extracellular vesicles (EVs) are lipid bilayer‐enclosed nanoparticles that are naturally released by almost all cell types into the extracellular space, where they transport proteins, lipids, and nucleic acids to facilitate intercellular communication and influence various physiological and pathological processes.^23^, ^24^ Notably, EVs secreted from the brain are present in the circulatory system, and these plasma‐circulating, brain‐derived EVs hold great promise as a non‐invasive source of AD biomarkers that reflect brain physiologic processes.^25^, ^26^ EVs enriched for neuronal origin offer a more sensitive and specific platform for biomarker discovery than bulk plasma or serum, as demonstrated by their capacity to enable phosphorylated tau (p‐tau)181 detection at concentrations that remain undetectable in circulating plasma using sensitive assays.^27^ Therefore, brain organoid‐secreted EV proteomic signatures could also offer molecular insights into disease progression, serve as preclinical models for drug efficacy tests, and assess patient response to therapeutic intervention strategies in clinical settings. Concurrently, leveraging iPSC‐derived brain organoids and EV‐based biomarkers represents a promising frontier in AD research. These innovative platforms bridge the gap between genetic risk and clinical presentation, offering new opportunities for mechanistic insight and personalized intervention.

To this end, we generated serotonergic (5‐HT) hindbrain organoids derived from 30 individuals, including both AD patients and cognitively normal controls. Because the hindbrain is the primary site of serotonergic neuron development,^28^ generating 5‐HT hindbrain organoids provides a physiologically relevant system to assess SSRI response and capture interindividual variability in drug response that mirrors clinical heterogeneity in AD. Therefore, through proteomic profiling of these organoids and their secreted EVs, we aim to identify molecular signatures associated with AD and treatment response to the SSRI escitalopram oxalate (EO), thus emphasizing the potential of this system for future precision medicine efforts in AD. This study represents the largest brain organoid cohort to date, involving 30 iPSC lines, and is also the first to concurrently generate molecular data from both organoids and their corresponding EVs.

**RESEARCH‐IN‐CONTEXT**

1. **Systematic review**: Previous models of Alzheimer's disease (AD), including animal and 2D cell cultures, have limitations in capturing human‐specific brain architecture and cellular diversity. Recent advances have introduced patient‐derived 3D brain organoids as promising platforms to model AD‐relevant pathology and drug responses more comprehensively.
2. **Interpretation**: Our study presents the largest cohort of serotonergic hindbrain organoids and their extracellular vesicles (EVs) from 30 individuals, demonstrating reproducible proteomic signatures relevant to AD. We identify shared and individual molecular alterations, highlighting the capacity of organoids and EVs to model disease heterogeneity and therapeutic response variability, including to selective serotonin reuptake inhibitors.
3. **Future directions**: This dual‐platform approach advances precision medicine by enabling scalable, patient‐specific drug screening and biomarker discovery. Further expansion of such models holds promise for improving clinical trial success and personalized interventions in AD.

## 2 METHODS

### 2.1 iPSC generation by peripheral blood mononuclear cell reprogramming

Patient‐derived iPSC lines were generated in house, as we have previously reported.^29^, ^30^ Briefly, healthy individuals without cognitive impairment (*n* = 6; Table 1) and AD patients (*n* = 24; Table 1) were recruited through the Johns Hopkins Alzheimer's Disease Research Center (ADRC), the Memory and Alzheimer's Treatment Center (MATC), or the Escitalopram for Agitation in Alzheimer's Disease (S‐CitAD) study.^31^, ^32^ AD patients were diagnosed with clinically defined AD using the Diagnostic and Statistical Manual of Mental Disorders 5th Edition criteria for major neurocognitive disorder due to AD.^33^ All participants provided informed consent under the oversight of a Johns Hopkins Institutional Review Board (NA_00045104) prior to blood sampling. Peripheral blood mononuclear cells (PBMCs) were isolated from patient blood, reprogrammed into iPSCs by transient expression of plasmid vectors through nucleofection, and cultured until passage 15 to 20, as reported previously.^29^ The quality of the generated iPSC lines was confirmed by karyotyping, flow cytometry, and immunocytochemistry for iPSC markers TRA‐160, OCT4, and NANOG (Figure S1 in supporting information). All experiments were conducted according to regulations governing the use of human stem cell lines in biomedical research.

**TABLE 1:** Clinical and demographic information of *n* = 30 study participants.

Sample Name	Patient Dx	Age	Sex	*APOE* genotype	Recruitment center	Batch
NCI‐1	NCI/Healthy	88	F	ε3/ε3	ADRC	1
NCI‐2	NCI/Healthy	84	F	ε3/ε3	ADRC	1
NCI‐3	NCI/Healthy	85	F	ε3/ε3	ADRC	1
NCI‐4	NCI/Healthy	79	M	ε3/ε3	ADRC	2
NCI‐5	NCI/Healthy	75	F	ε3/ε3	ADRC	2
NCI‐6	NCI/Healthy	84	F	ε3/ε4	ADRC	2
AD‐1	AD	72	M	ε4/ε4	MATC	1
AD‐2	AD + NPS	70	M	ε3/ε4	S‐CitAD	1
AD‐3	AD + NPS	73	M	ε4/ε4	S‐CitAD	1
AD‐4	AD + NPS	98	F	ε3/ε4	S‐CitAD	2
AD‐5	AD + NPS	93	M	ε3/ε4	S‐CitAD	2
AD‐6	AD + NPS	93	F	ε3/ε3	S‐CitAD	2
AD‐7	AD + NPS	75	F	ε3/ε4	S‐CitAD	2
AD‐8	AD + NPS	83	M	ε3/ε4	S‐CitAD	2
AD‐9	AD + NPS	83	M	ε3/ε4	S‐CitAD	2
AD‐10	AD	70	F	ε3/ε3	ADRC	2
AD‐11	AD	64	M	ε3/ε4	ADRC	2
AD‐12	AD	62	F	ε3/ε3	ADRC	2
AD‐13	AD	57	F	ε3/ε3	MATC	2
AD‐14	AD	60	F	ε3/ε4	MATC	2
AD‐15	AD	58	F	ε3/ε4	MATC	2
AD‐16	AD	71	M	ε4/ε4	MATC	2
AD‐17	AD	65	F	ε3/ε4	MATC	2
AD‐18	AD	73	M	ε3/ε4	MATC	2
AD‐19	AD + NPS	79	M	ε3/ε4	S‐CitAD	2
AD‐20	AD + NPS	79	M	ε3/ε4	S‐CitAD	2
AD‐21	AD + NPS	92	F	ε3/ε3	S‐CitAD	2
AD‐22	AD	77	M	ε3/ε4	MATC	2
AD‐23	AD	78	F	ε3/ε3	MATC	2
AD‐24	AD + NPS	74	M	ε4/ε4	S‐CitAD	2

Abbreviations: AD, Alzheimer's disease; ADRC, Alzheimer's Disease Research Center; *APOE*, apolipoprotein E; Dx, diagnosis; F, female; M, male; MATC,  Memory and Alzheimer's Treatment Center; NCI, no cognitive impairment; NPS, neuropsychiatric symptoms; S‐CitAD, Escitalopram for Agitation in Alzheimer's Disease Study.

### 2.2 iPSC differentiation into 5‐HT organoids

Differentiation of iPSCs into 5‐HT organoids was performed using our established methodology.^9^ In brief, embryoid bodies (EBs) were formed by centrifugation to mimic early brain development. EBs were cultured and differentiated into neuroectoderm lineage, which, over the course of 6 to 8 weeks, were then patterned to generate the serotonin‐producing neuronal cells of interest. Eight organoids per well were kept in 6‐well plates with the appropriate media (2.5 mL/well). A detailed table with media composition at every stage of the differentiation process can be found in the supplementary information of our previously published methods.^9^

### 2.3 EO exposure

Organoids were either treated with 100 uM of EO for 1 week, or remained untreated, as we have reported previously.^9^ EO dosage was selected in accordance with prior studies that have evaluated cytotoxicity and measured serotonin release from iPSC‐derived neurons and organoids in the presence of EO at various concentrations.^9^, ^28^ On day 7, organoids were carefully washed with phosphate‐buffered saline (PBS), collected in 1.5 mL centrifugation tubes, and stored dry at −80°C before being sent to Tymora Analytical Operations (West Lafayette, IN) for proteomics analysis.

### 2.4 Separation and characterization of EVs

EVs were separated by repurposing our previously reported protocol.^34^, ^35^, ^36^, ^37^ Briefly, supernatant collected from each patient‐derived organoid underwent differential centrifugation steps (300 x g for 10 minutes, 2000 x g for 10 minutes, at 4°C) to remove cell debris. The supernatant was concentrated to a maximum of 500 µL through ultrafiltration (Amicon, RC, 100 kDa cutoff), and the filtrates were then loaded on a size exclusion chromatography (SEC) column and eluted with Dulbecco's PBS (DPBS). The supernatant was concentrated to a maximum of 500 µL through ultrafiltration (Amicon, RC, 100 kDa cutoff), and the filtrates were then loaded on a size exclusion chromatography (SEC) column and eluted with DPBS, separating EVs from co‐isolated free proteins and aggregates. The first four fractions (2 mL) containing purified EVs were pooled and concentrated by ultrafiltration (Amicon, RC, 10 kDA cutoff) to a final volume of 200 µL, and protein quantification was performed using a bicinchoninic acid assay (Pierce BCA Protein Assay Kit, Thermo Fisher Scientific). Purified EVs were characterized by nano flow cytometry (nanoFCM; Figure S2A in supporting information), single‐particle interferometric reflectance imaging sensor (SP‐IRIS; Figure S2B), and transmission electron microscopy (TEM; Figure S2C) or sent to Tymora Analytical Operations (West Lafayette, IN) for proteomics analysis.

### 2.5 Preparation of organoid proteomics samples

Standard proteomics sample preparation was performed by Tymora. For organoids, the full volume of each organoid sample was lysed and denatured to extract proteins using the phase‐transfer surfactant (PTS)‐aided procedure.^38^ The proteins were reduced and alkylated by incubation in 10 mM tris(2‐carboxyethyl)phosphine (TCEP) and 40 mM chloroacetamide (CAA) for 10 minutes at 95°C. The extracted proteins were captured onto 40 µL of magnetic beads using a one‐pot procedure, as described previously,^39^ by adding acetonitrile to 70% final concentration and incubating 10 minutes at room temperature. The beads were then washed three times with 70% acetonitrile and excess acetonitrile solution was allowed to dry. The proteins were recovered by addition of 50 mM tetraethylammonium bromide, and the protein concentration detected by BCA assay. The proteins were digested with Lys‐C (Wako) at 1:100 (w/w) enzyme‐to‐protein ratio for 1 hour at 37°C in 50 µL of 50 mM triethyl ammonium bicarbonate. Trypsin was added to a final 1:50 (w/w) enzyme‐to‐protein ratio for 3 hours of digestion at 37°C. To recover the peptides, acetonitrile was added to each sample to a final concentration of 60% to capture the enzymes onto beads. The peptide samples were recovered in the supernatant and dried completely in a vacuum centrifuge and stored at −80°C. A portion of each sample was used for peptide quantitation using Pierce Colorimetric Peptide Quantitation Kit (Thermo Fisher).

### 2.6 Preparation of full EV proteomics samples

Preparation of full EV proteomics was performed using the same methods as for the organoid procedure above, except that the extracted proteins from each EV sample were captured onto 20 µL of magnetic beads.

### 2.7 Liquid chromatography‐tandem mass spectrometry analysis

Standard liquid chromatography‐tandem mass spectrometry (LC‐MS/MS) analysis was performed by Tymora, as outlined previously,^40^ except that peptide samples were dissolved at 0.1 µg/µL concentration in 0.05% trifluoroacetic acid with 3% (vol/vol) acetonitrile, and 5 µL of each sample was injected into an Ultimate 3000 nano Ultra‐High Performance Liquid Chromatography (UHPLC) system (Thermo Fisher Scientific). The mobile phase buffer was run with a linear 90‐minute gradient of 6% to 30% buffer B, and the UHPLC was coupled online with an Exploris 480 mass spectrometer (Thermo Fisher Scientific). The mass spectrometer was operated in data‐dependent mode, beginning with a full‐scan MS (from m/z 375 to 1200 at a resolution of 60,000), followed by MS/MS of the 30 most intense ions. MS/MS settings included a resolution of 15,000, normalized collision energy set at 30%, normalized automatic gain control target (AGC) at 50%, maximum injection time of 30 ms, and a 60 second exclusion period.

### 2.8 LC‐MS data processing

Raw data files were searched against a non‐redundant human protein database (updated in 2024) using the Byonic (Protein Metrics) and Sequest search engines integrated within Proteome Discoverer 3.1 (Thermo Fisher Scientific). The MS1 precursor mass tolerance was set to 10 ppm, and the MS2 fragment ion tolerance to 20 ppm. Search parameters included static modification of cysteine residues by carbamidomethylation (+57.0214 Da), with variable modifications allowing for methionine oxidation (+15.9949 Da) and N‐terminal protein acetylation (+42.011 Da). Trypsin/P was specified as the digestion enzyme, permitting up to two missed cleavages per peptide. The false discovery rate (FDR) threshold was set at 1% for both peptide and protein identifications. Identified proteins and peptides were grouped, redundant entries were excluded, and only unique peptides and unique master proteins were reported.

### 2.9 Label‐free quantitation analysis

All data were quantified using the label‐free quantitation node of Precursor Ions Quantifier through the Proteome Discoverer v3.1 (Thermo Fisher Scientific). For the quantification of proteomic data, the intensities of peptides were extracted with initial precursor mass tolerance set at 10 ppm, minimum number of isotope peaks as 2, maximum ΔRT of isotope pattern multiplets–0.2 minutes, peptide‐spectrum match (PSM) confidence FDR of 0.01, with hypothesis test of analysis of variance, maximum retention time shift of 5 minutes, pairwise ratio‐based ratio calculation, and 100 as the maximum allowed fold‐change. For calculations of fold change between the groups of proteins, total phosphoprotein abundance values were added, and the ratios of these sums were used to compare proteins within different samples.

### 2.10 Bioinformatics analyses

To retain the characteristics of the original data as much as possible and reduce the FDR, we did not perform any missing value imputation during data preprocessing. After obtaining the protein abundance data from both experimental batches, we used the HarmonizR (version 1.4.0) R package based on the ComBat algorithm for batch effect correction (BEC).^41^, ^42^ Compared to the traditional BEC methods, HarmonizR is specifically designed for proteomic data and can effectively handle proteomic data containing missing values without imputations. This approach preserves the intrinsic biological variance of the data while minimizing the introduction of artificial variability associated with imputation‐based methods. Batch correction was performed independently of disease status, ensuring that group‐specific biological signals were not introduced, distorted, or attenuated during the correction process and thereby maintaining the integrity of downstream differential expression and EO‐response analyses. Subsequently, we applied the modified 80% rule,^43^, ^44^ retaining only proteins that met any of the following conditions: (1) detected in > 80% of AD samples without EO treatment, (2) detected in > 80% of AD samples with EO treatment, (3) detected in > 80% of no cognitive impairment (NCI) samples without EO treatment, or (4) detected in > 80% of NCI samples with EO treatment. The remaining proteins were subsequently subjected to median normalization and log_2_ transformation. This post‐correction filtering and normalization strategy reduces noise arising from low coverage features and mitigates potential artifacts introduced during batch effect correction, thereby improving the statistical robustness of downstream analyses. Then, we performed an unpaired two‐tailed Student *t* test between the EV and organoid groups, as well as between the AD and NCI groups. For the comparison between the EO treatment and control groups, we performed a paired two‐tailed Student *t* test. The resulting *P* values were adjusted using the Benjamini–Hochberg (BH) method to control the FDR, and only proteins with *P*adj < 0.05 and |log_2_FC| > 1 were considered significantly differentially expressed (DE). To visualize the expression differences between groups, we plotted principal component analysis (PCA) plots for all proteins, as well as for significantly DE proteins (DEPs) only, using the ggplot2 (version 3.5.2) R package. We also created the heatmap for only DEPs with pheatmap (version 1.0.12) R package, and the volcano plots for all proteins with ggplot2 (version 3.5.2) and ggrepel (version 0.9.6) packages.

To look for interactions between the DEPs, we submitted our lists of DEPs (Tables S1–S4 in supporting information) to the STRING database of protein–protein interactions (string‐db.org),^45^, ^46^ using default settings. To preserve the integrity of the reported network enrichment *P* values, we limited our analysis to direct interactions only, without adding additional interacting partners. STRING also performs functional enrichment analyses of Gene Ontology (GO)^47^ from AmiGO2 (https://amigo.geneontology.org/amigo), pathways from the Kyoto Encyclopedia of Genes and Genomes (KEGG; https://www.kegg.jp/kegg/pathway.html) and Reactome (https://reactome.org/), and annotated keywords from UniProt (https://www.uniprot.org/).

To assess how DEPs may be associated with AD risk, for proteins that were DE in AD‐derived organoids and EVs, we queried their corresponding protein‐coding genes in the AD gene discovery database, Agora (https://agora.adknowledgeportal.org Tables S1–S4). The Agora database provides information on expression quantitative trait loci (eQTLs) in disease‐relevant brain tissue, whether the corresponding RNA or protein has been previously identified as a DE molecule in the AD brain, and whether the gene is associated with late‐onset AD (LOAD) or is implicated as a potential therapeutic target.^48^ Agora also assigns each gene a genetic risk score, which integrates evidence from multiple genetic studies to summarize the strength of its association with LOAD. Scores range from 0 to 3, with higher values indicating stronger genetic support for the gene's involvement in disease risk, as previously described.^49^

## 3 RESULTS

### 3.1 Joint processing strategy reduces batch effects and preserves biological signal

As a proof of concept, we first generated 5‐HT hindbrain organoids from three healthy individuals without cognitive impairment (NCI‐1, NCI‐2, NCI‐3) and three patients diagnosed with AD (AD‐1, AD‐2, AD‐3). Each of the six lines (Table 1) was cultured in triplicate, and proteomics data were acquired from both the organoids and their corresponding EVs. After confirmation that we could reliably generate 5‐HT organoids and obtain high‐quality proteomics data from both organoids and EVs, we expanded the study by generating three additional NCI‐derived organoids (NCI‐4, NCI‐5, NCI‐6) and 21 additional AD‐derived organoids (AD‐4 to AD‐24). To optimize time and resources, we opted to culture and analyze these 24 lines (Table 1) as singular samples rather than in triplicate, providing greater sample diversity and improved resolution for precision medicine applications.

As expected, this experimental design introduced noticeable batch effects between the initial six triplicate samples and the subsequent 24 singular samples. These batch effects were apparent in both the organoid and EV proteomic datasets and likely reflect differences in timing, sample handling, and subtle variations in culture conditions or processing protocols across batches. While the triplicate design of the initial samples allowed us to assess technical reproducibility, the larger cohort provided broader biological insights at the expense of introducing greater variability.

To mitigate batch effects and ensure meaningful comparisons across the full dataset, we used two parallel correction strategies: processing each batch independently and processing all samples jointly. Both approaches effectively reduced batch‐associated variability; however, analyzing the first batch (NCI‐1, NCI‐2, NCI‐3, AD‐1, AD‐2, AD‐3) separately introduced a confounding variable due to the inclusion of only female NCI participants and male AD participants in this batch of samples. This imbalance led to significant DE of sex‐specific proteins, which would complicate the interpretation of disease‐related effects (AD vs. NCI and EO vs. control) by potentially conflating sex differences with disease status.

**FIGURE 1:**
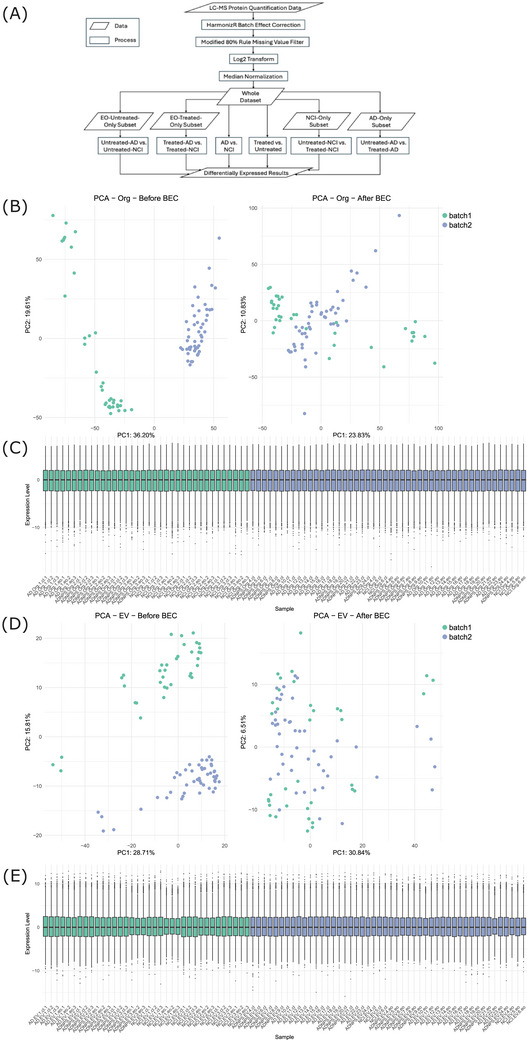
Data processing, BEC, and normalization. A, The bioinformatics pipeline used to correct batch effects, apply missing value filters, and perform log2 normalization to organoids and EVs from batch 1 and batch 2, prior to DE analysis. B, PCAs of organoid samples from batch 1 and batch 2 demonstrate that our processing pipeline was able to mitigate batch effects and (C) normalize protein expression levels across batches. D, PCAs of EV samples from batch 1 and batch 2 demonstrate that our processing pipeline was able to mitigate batch effects and (E) normalize protein expression levels across batches. AD, Alzheimer's disease; BEC, batch effect correction; DE, differential expression; EO, escitalopram oxalate; EV, extracellular vesicle; NCI, no cognitive impairment; PC, principal component; PCA, principal component analysis.

We then performed batch correction, missing value filtering, median normalization, and log_2_ transformation methods jointly across all samples (Figure 1A). To preserve inter‐individual variation in expression, we did not impute missing values during data processing. Instead, prior to applying standard median normalization and log_2_ transformation,^41^, ^42^ we implemented the modified 80% rule^43^, ^44^ as a stringent filtering step. This rule retained only those proteins detected in at least 80% of samples within any one treatment group (AD without EO treatment, AD with EO treatment, NCI without EO treatment, or NCI with EO treatment). This approach was chosen to avoid introducing artificial signals through imputation while ensuring that features were consistently detected in biologically meaningful subgroups. It also preserves signals that may be specific to certain disease or treatment states, which are often lost using more conservative global filtering approaches. The resulting dataset effectively mitigated batch effects and ensured consistent normalization across both organoid and EV datasets (Figure 1B–E). By applying these strict filtering criteria, we were also able to confidently analyze biologically meaningful patterns despite the high degree of inter‐individual variability introduced by using 30 individual cell lines.

### 3.2 5‐HT organoids and EVs represent distinct sources of biological information that exhibit internally consistent expression profiles

To demonstrate that our differentiation protocol could reliably generate 5‐HT hindbrain organoids across 30 individuals from AD and NCI backgrounds, as well as confirm the quality of proteomics data obtained from both organoids and their corresponding EVs, we first examined the expression of key cell‐specific marker proteins across all samples.

**FIGURE 2:**
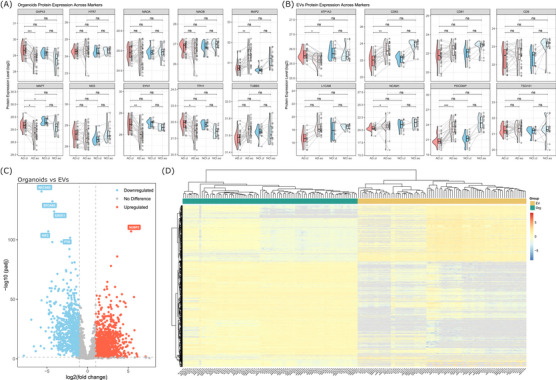
Characterization of neuronal organoids and neuronal‐derived EVs. A, Violin plots showing the log_2_ expression of key neuronal and serotonergic protein markers among organoids derived from AD and NCI participants. B, Violin plots showing the log_2_ expression of key EV and neuronal‐derived markers among EVs derived from AD and NCI participants. Using a paired two‐tailed Student *t* test statistical comparisons were performed on median‐normalized values; however, to visualize relative expression levels across samples, violin plots display batch‐corrected, log_2_‐transformed protein expression levels prior to median normalization. C, Volcano plot showing the log_2_ fold change and ‐log_10_(*P* value) of protein expression between organoid and EV samples. Red = DEPs with *P*adj < 0.05 and log_2_FC > 1; Blue = Red = DEPs with *P*adj < 0.05 and log_2_FC < 1, using a paired two‐tailed Student *t* test. D, Heatmap showing the distinct clustered separation of organoid and EV samples. Sample classification structure: Diagnosis.SampleType.BiologicalReplicate.Treatment.TechnicalReplicate (e.g., NCI.EV.1.cl.1 = No Cognitive Impairment, Extracellular Vesicle, Biological Replicate 1, Control, Technical Replicate 1). AD, Alzheimer's disease; DEP, differentially expressed protein; EV, extracellular vesicle; NCI, no cognitive impairment; Org, organoid.

As expected, we observed consistent expression of neuronal markers across our organoid samples (Figure 2A). When comparing organoids derived from AD patients to healthy cognitive controls, our organoid samples expressed non‐significant differences in the expression of proteins indicative of maturing neurons, including growth associated protein 43 (GAP43; *P*adj = 0.6624), microtubule‐associated protein tau (MAPT; *P*adj = 0.1864), microtubule‐associated protein 2 (MAP2; *P*adj = 0.5817), nestin (NES, *P*adj = 0.9656), synapsin I (SYN1; *P*adj = 0.1535), and β‐tubulin III (TUBB3; padj = 0.6316). We also observed non‐significant differences in expression levels of 5‐HT neuronal markers 5‐hydroxytryptamine receptor 7 (HTR7; *P*adj = 0.9947), monoamine oxidase A (MAOA; *P*adj = 0.6449), monoamine oxidase B (MAOB; *P*adj = 0.3954), and tryptophan hydroxylase 1 (TPH1; *P*adj = 0.8355).

However, between‐group comparisons suggested that EO exposure was responsible for significant changes in the expression of GAP43 (*P*adj = 0.0002), MAP2 (*P*adj = 0.0049), MAPT (*P*adj = 0.0235), SYN1 (*P*adj = 0.0046), and TPH1 (*P*adj = 0.0398), but only comparing AD organoids with EO treatment to AD organoids without EO treatment (Figure 2A). There were no observable effects of EO exposure across NCI organoids. Ultimately, these results highlight the scalability and high reproducibility of our approach for generating 5‐HT neuronal organoids.

We performed analogous comparisons of the corresponding EV samples to evaluate the presence of canonical EV markers and neuron‐specific EV proteins, aiming to confirm their identity and cellular origin. When querying the top five EV‐associated proteins identified by Vesiclepedia (http://microvesicles.org/extracellular_vesicle_markers)^23^ —CD63, CD9, programmed cell death 6 interacting protein (PDCD6IP), tumor susceptibility 101 (TSG101), and CD81—we observed that these markers were not significantly DE between EVs from AD‐ or NCI‐derived organoids (Figure 2B). Based on characterization of EVs by SP‐IRIS (Figure S2), these results aligned with our expectations that there should be no significant differences between AD‐ and NCI‐derived EVs in the expression of canonical EV tetraspanins CD9 (*P*adj = 0.2390), CD63 (*P*adj = 0.1756), and CD81 (*P*adj = 0.2818), as well as other common EV markers, PDCD6IP (*P*adj = 0.4967) and TSG101 (*P*adj = 0.7720).

We also queried markers indicative of neuronal‐derived EVs (NDEVs), including ATPase Na+/K+ Transporting subunit alpha 3^50^ (ATP1A3; *P*adj = 0.1033), L1 cell adhesion molecule^51^ (L1CAM *P*adj = 0.3527), and neural cell adhesion molecule 1^51^ (NCAM1; *P*adj = 0.0898), and confirmed that these markers were also not significantly DE between AD‐ and NCI‐derived samples (Figure 2B). Surprisingly however, L1CAM and NCAM1 were not consistently detected across all EV samples—50% of AD‐control, 26.6% of AD‐EO, 25% of NCI‐control, and 16.6% of NCI‐EO samples were missing values for L1CAM; while 36.6% of AD‐control, 6.6% of AD‐EO, and 16.6% of NCI‐control samples were missing values for NCAM1. Although previous studies have identified L1CAM and NCAM1 as surface markers enriched on NDEVs,^26^, ^51^ our results add to an emerging body of work suggesting that these proteins may not fully distinguish NDEVs from EVs derived from other cell types.^52^, ^53^ When we assessed the expression of these EV and NDEV markers, we again observed that while there were no significant differences in expression levels between AD and NCI EVs, ATP1A3 (*P*adj = 0.0337), CD63 (*P*adj = 0.0056), NCAM1 (*P*adj = 0.0257), and PDCD6IP (*P*adj < 0.0001) were significantly DE in EO‐treated AD‐derived EVs compared to untreated AD‐derived EVs (Figure 2B), but not between the NCI EVs. Akin to the effect of EO on hindbrain organoids, only EVs from AD patients exhibited significant changes in the expression of key marker proteins, while these key markers remain at similar levels between treated and untreated NCI EVs.

Finally, to further validate that the hindbrain organoids and their corresponding EVs represent biologically distinct sources of biological information, we performed DE analysis and visualized the top DEPs using both a volcano plot and a heatmap. These analyses clearly demonstrate that the organoid and EV samples have distinct proteomic profiles (Figure 2C and 2D), supporting the conclusion that they represent distinct systems. Overall, we demonstrate that our 5‐HT hindbrain organoids consistently generate NDEVs. Importantly, while derived from the same source, the organoids and their corresponding EVs represent biologically distinct but complementary model systems, each capturing unique aspects of AD pathogenesis.

### 3.3 Differential protein expression profiles in 5‐HT organoids capture elevated AD risk

**FIGURE 3:**
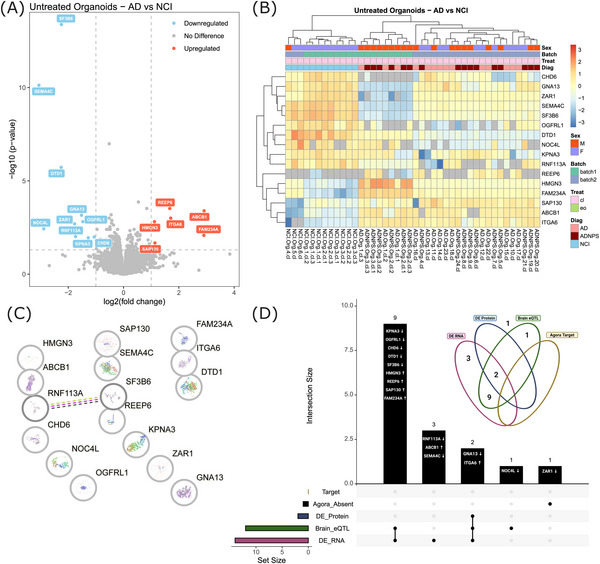
Comparative proteomic profiling of 5‐HT hindbrain organoids derived from AD patients and controls with NCI. A, A volcano plot showing the log_2_ fold change and ‐log_10_(*P* value) of protein expression in untreated (control) organoids from AD patients, relative to organoids from healthy participants with NCI. Red = DEPs with *P*adj < 0.05 and log_2_FC > 1; Blue = Red = DEPs with *P*adj < 0.05 and log_2_FC < 1, using a paired two‐tailed Student *t* test. All 16 DEPs are labelled. B, A heatmap showing proteomics expression profiles of the 16 DEPs between AD‐organoids and NCI‐organoids shows that protein expression profiles cluster organoids based on disease status. Sample classification structure: Diagnosis.SampleType.BiologicalReplicate.Treatment.TechnicalReplicate (e.g., NCI.Org.4.cl = No Cognitive Impairment, Organoid, Biological Replicate 4, Control); however, (C) using a hypergeometric test with FDR correction, STRING analysis did not indicate significant GO or pathway enrichments among the 16 DEPs. D, An upset plot and Venn diagram showing how the 16 DEPs are represented in the Agora database (Target = Predicted therapeutic target in Agora Database; Agora_Absent = absent from Agora Database; DE_protein = Known Differentially Expressed protein in AD; Brain_eQTL = Known Brain expression Quantitative Trait Loci in AD; DE_RNA = Known Differentially Expressed RNA transcript in AD). 5‐HT, serotonergic; AD, Alzheimer's disease; cl, control; DEP, differentially expressed protein; Diag, diagnosis; EO, escitalopram oxalate; FDR, false discovery rate; GO, Gene Ontology; NCI, no cognitive impairment; NPS, neuropsychiatric symptoms; Treat, treatment.

To investigate whether molecular differences in 5‐HT organoids might serve as potential biomarkers of AD risk, we next examined protein expression profiles in untreated (control) organoids derived from both AD and NCI participants. Due to the immense patient genetic variability reflected in the organoids, we used very strict filtering criteria to identify DEPs that reflect the most biologically relevant differences driving the origin of AD in these samples. In addition to using the modified 80% rule in our data filtration step, we elected to call a protein significantly DE if it met a threshold of *P*adj < 0.05 and |log_2_FoldChange| > 1. Using these filters, only 16 DEPs were identified in AD organoids compared to NCI organoids: 6 were significantly upregulated, and 10 downregulated (Figure 3A; Table S1). We generated a heatmap of these DEPs in AD and NCI organoids and observed that the proteomic profiles of AD and NCI (“Diag” = light blue) samples clustered according to diagnosis (Figure 3B) but were unable to distinguish AD patients (AD‐“Diag” = light pink) without NPSs from those with NPSs (AD+NPS‐“Diag” = dark red).

We used the STRING database of protein–protein interactions^45^, ^46^ to test whether these 16 DEPs are functionally related, as evidenced by reported interactions between them, and complemented this analysis with functional enrichment analyses to identify potentially overrepresented pathways, biological processes, molecular functions, or cellular components. STRING (Figure 3C) determined that there are no significant interactions or GO enrichments between or among these proteins. Therefore, to assess the potential disease relevance of these DEPs—particularly their association with AD risk—we next queried each corresponding protein‐coding gene in the AD gene discovery database Agora (https://agora.adknowledgeportal.org). Of the 16 DEPs, all but one (ZAR1; variants in which have been previously implicated in elevated AD risk^54^) are encoded by known brain eQTLs or have corresponding RNA transcripts known to be significantly DE in at least one brain region (Figure 3D; Table S1), based on work conducted by the National Institute on Aging's Accelerating Medicines Partnership for Alzheimer's Disease (AMP‐AD) Consortium.^55^

For example, coiled‐coil‐helix‐coiled‐coil‐helix domain containing 6 (CHCHD6) was significantly downregulated in our AD organoids (*P*adj < 0.001), consistent with reports linking CHCHD6 reduction to enhanced amyloid precursor protein (APP) processing, mitochondrial dysfunction, and amyloid pathology. Downregulation of D‐aminoacyl‐tRNA deacylase 1 (DTD1; *P*adj < 0.0001) in our dataset is also consistent with evidence that DTD1 plays an essential role in regulating dendritic spine density and synaptic structure of hippocampal pyramidal neurons, as well as spatial learning and memory performance in rodent models.^56^ Likewise, downregulation/disruption of semaphorin 4C (SEMA4C; *P*adj < 0.0001), which has functional implications involving synaptic plasticity, attenuation of neuronal apoptosis, and neuroprotection,^57^ leads to impaired ability to retain both recent and remote fear memories in AD models.^58^

In addition to implicating previously known AD‐relevant DEPs, the Agora database also assigns each gene a genetic risk score, which integrates evidence from multiple genetic studies to summarize the strength of its association with AD. Scores range from 0 to 3, with higher values indicating greater involvement in mediating disease risk (e.g., apolipoprotein E = 2.85).^49^ The average Agora genetic risk score for the genes corresponding to our set of DEPs was 1.3367 ± 0.4369. These results suggest that the DEPs in our 5‐HT organoids represent dysregulated gene products that are known to elevate AD risk; however, they do not appear enriched in common pathways or biological processes.

### 3.4 Differential protein expression profiles in EVs captures AD‐relevant biology and may represent a reliable source of biomarkers for AD risk

**FIGURE 4:**
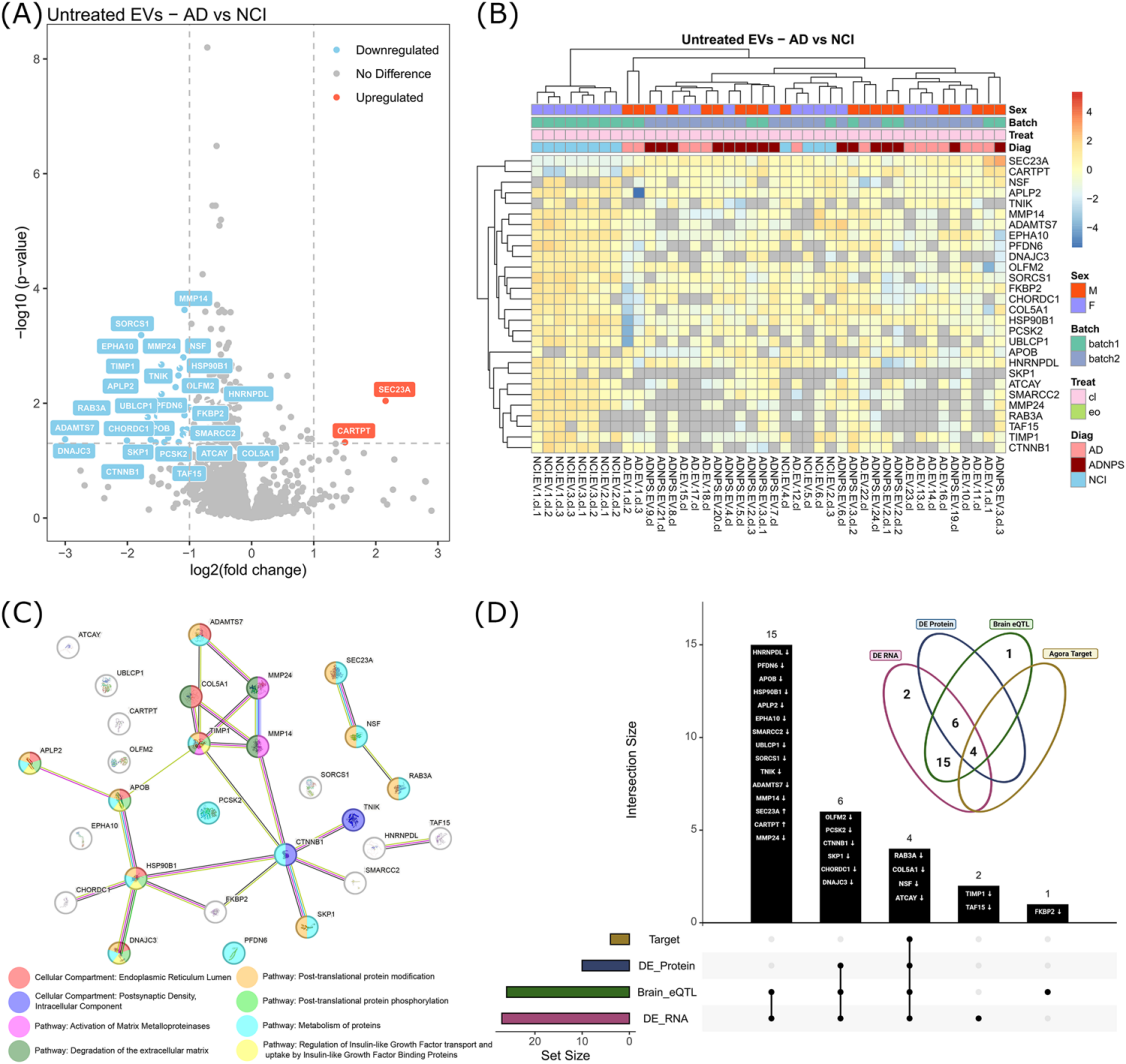
Comparative proteomic profiling of EVs released from AD‐organoids and NCI‐organoids. A, A volcano plot showing the log_2_ fold change and ‐log_10_(*P* value) of protein expression in EVs released from untreated AD‐organoids, relative to EVs released from untreated NCI‐organoids. Red = DEPs with *P*adj < 0.05 and log_2_FC > 1; Blue = Red = DEPs with *P*adj < 0.05 and log_2_FC < 1, using a paired two‐tailed Student *t* test. All 28 DEPs are labelled. B, A heatmap showing proteomics expression profiles of the 28 DEPs between AD‐organoids and NCI‐organoids shows that protein expression profiles mostly cluster EVs based on disease status, with some exceptions. Sample classification structure: Diagnosis.SampleType.BiologicalReplicate.Treatment.TechnicalReplicate (e.g., NCI.EV.1.cl.1 = No Cognitive Impairment, Extracellular Vesicle, Biological Replicate 1, Control, Technical Replicate 1). C, Using a hypergeometric test with FDR correction, STRING analysis revealed significant GO and pathway enrichments among the 28 DEPs. D, An upset plot and Venn diagram showing how the 28 DEPs are represented in the Agora database (Target = Predicted therapeutic target in Agora Database; Agora_Absent = absent from Agora Database; DE_protein = Known Differentially Expressed protein in AD; Brain_eQTL = Known Brain expression Quantitative Trait Loci in AD; DE_RNA = Known Differentially Expressed RNA transcript in AD). AD, Alzheimer's disease; cl, control; DEP, differentially expressed protein; Diag, diagnosis; EO, escitalopram oxalate; EV, extracellular vesicle; FDR, false discovery rate; GO, Gene Ontology; NCI, no cognitive impairment; NPS, neuropsychiatric symptoms; Treat, treatment.

Next, to investigate whether we could use EVs to identify molecular differences that could serve as potential AD biomarkers, we used the same strict thresholds to examine protein expression profiles from the EVs released from the untreated (control) AD and NCI organoids. In EVs, 28 DEPs were identified by using the same strict thresholds (modified 80% rule, *P*adj < 0.05, |log2FoldChange| > 1). Two of the DEPs were significantly upregulated, and 26 were significantly downregulated in AD EVs compared to NCI EVs (Figure 4A; Table S2). We generated a heatmap of these DEPs in AD and NCI EVs, and to our surprise, we observed that while most NCI‐EV samples from batch 1 clustered together, all NCI‐EV samples from batch 2 clustered with one NCI replicate from batch 1 and two AD samples from batch 2 (Figure 4B). Again, the proteomic profiles of EVs were unable to distinguish AD from AD+NPS.

STRING analysis showed that the 28 DEPs between AD‐EVs and NCI‐EVs are highly interconnected (STRING enrichment, *p* = 7.35 × 10^−5^) with 2.56‐fold more interconnections than expected by chance for the same number of proteins. Functional enrichment analysis for the network proteins showed enrichment for 22 terms (Table S2) including GO enrichment for cellular components “endoplasmic reticulum lumen” (FDR = 0.0005) and “postsynaptic density, intracellular component” (FDR = 0.044), as well as KEGG pathway enrichment for “protein processing in endoplasmic reticulum” (FDR = 0.0291) and reactome pathway enrichment for “post‐translational protein phosphorylation” (FDR = 0.0011), “activation of matrix metalloproteinases” (FDR = 0.0081), and “metabolism of proteins” (FDR = 0.0011; Figure 4C).

We again queried each of the corresponding protein‐coding genes in the Agora database and found that all 28 DEPs are encoded by known brain eQTLs or have corresponding RNA transcripts known to be significantly DE in at least one brain region. Furthermore, among our set of DEPs, 10 are already known DEPs in the AD brain, and four of these (Ras‐related protein Rab‐3A [RAB3A],^59^, ^60^ abstract background collagen type V alpha 1 chain [COL5A1],^61^ *N*‐ethylmaleimide‐sensitive factor [NSF],^60^ and ATCAY kinesin light chain interacting caytaxin [ATCAY]^62^) are present on a list of nominated genes that have been identified as putative targets for new AD treatment or prevention, as contributed to Agora by researchers from the AMP‐AD consortium (Figure 4D; Table S2).

The average Agora genetic risk score for the genes corresponding to this set of DEPs was 1.4846 ± 0.4975, again suggesting that our AD 5‐HT organoids, and the EVs they secrete, can capture modestly elevated AD risk, compared to the EVs released by NCI organoids. Overall, these results suggest that the DEPs in the EVs released by our 5‐HT organoids can distinguish disease status between AD and NCI and even reveal dysregulation of nominated AD target genes.

### 3.5 Differential protein expression profiles in 5‐HT organoids treated with EO reveals patient heterogeneity in drug response

We next sought to determine whether these models could also serve as effective platforms for drug screening of a potentially therapeutic compound in a precision medicine context. To this end, we treated organoids from all 30 individuals with the SSRI EO. Our goal was to assess whether EO exposure induces measurable and DE changes in protein expression profiles, thereby providing an early indication of drug responsiveness at the molecular level.

**FIGURE 5:**
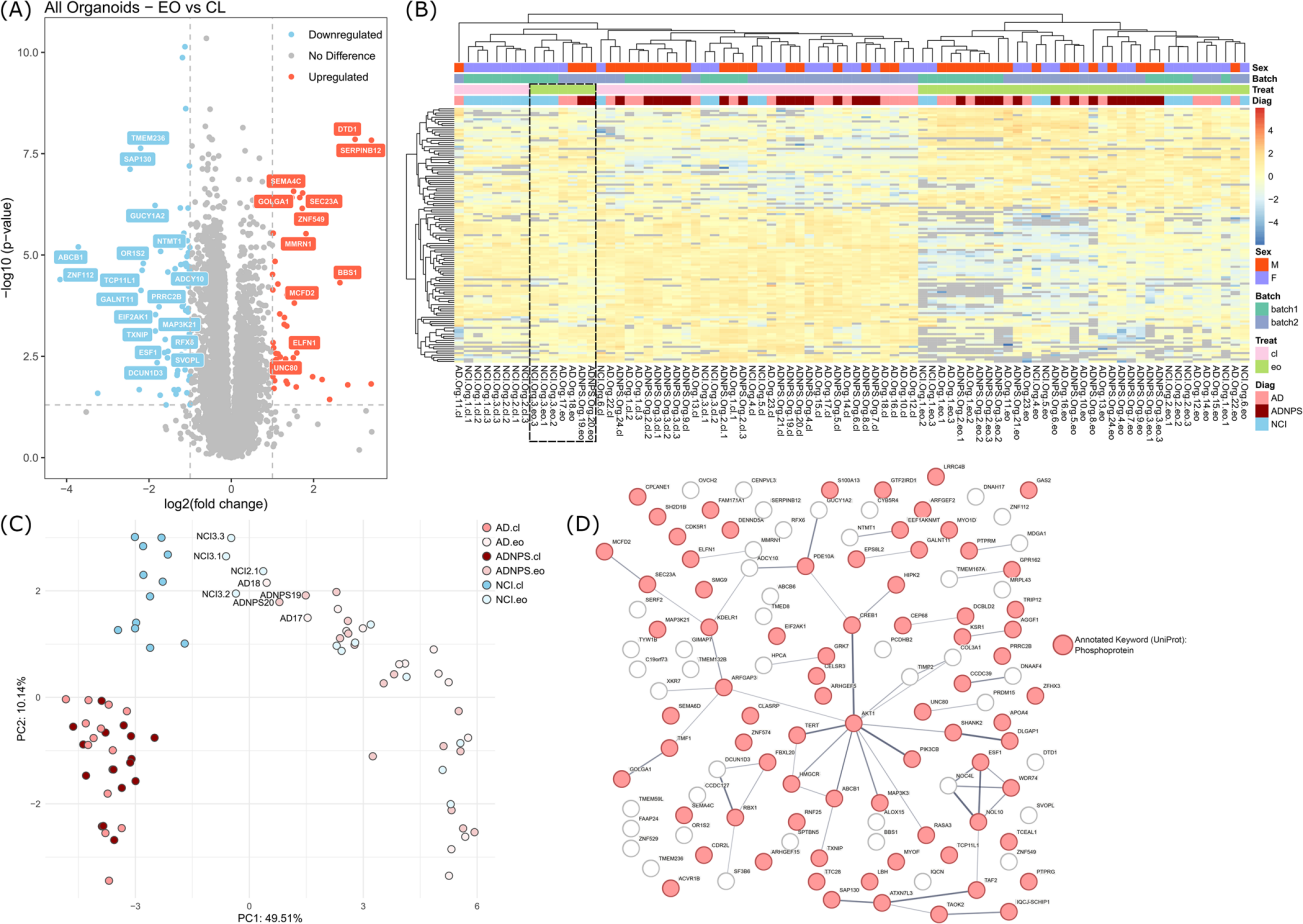
Comparative proteomic profiling of EO‐treated 5‐HT organoids and no treatment control organoids. A, A volcano plot showing the log_2_ fold change and ‐log_10_(*P* value) of protein expression in organoids treated with EO, relative to untreated (control; CL) organoids. Red = DEPs with *P*adj < 0.05 and log_2_FC > 1; Blue = Red = DEPs with *P*adj < 0.05 and log_2_FC < 1, using a paired two‐tailed Student *t* test. Labeled proteins = *P*adj < 0.01 and |log_2_FC| > 1.5. B, A heatmap showing proteomics expression profiles of the 121 DEPs between EO‐treated and untreated organoids. Sample classification structure: Diagnosis.SampleType.BiologicalReplicate.Treatment.TechnicalReplicate (e.g., NCI.EV.1.cl.1 = No Cognitive Impairment, Extracellular Vesicle, Biological Replicate 1, Control, Technical Replicate 1). C, PCA demonstrates that EO‐treated and control organoids cluster independently, but that a subset of EO‐treated organoids may be unresponsive to the drug. D, Using a hypergeometric test with FDR correction, STRING analysis did not yield significant pathway enrichments among the 121 DEPs; however, there was a significant enrichment of UniProt keyword “Phosphoprotein” among DEPs (red). 5‐HT, serotonergic; AD, Alzheimer's disease; cl, control; DEP, differentially expressed protein; Diag, diagnosis; EO, escitalopram oxalate; FDR, false discovery rate; NCI, no cognitive impairment; NPS, neuropsychiatric symptoms; PCA, principal component analysis; Treat, treatment.

Again, by using a threshold of *P*adj < 0.05 and |log_2_FoldChange| > 1 in our filtered dataset, we were able to detect 121 DEPs, 47 of which were significantly upregulated and 74 of which significantly downregulated in EO‐treated organoids compared to control (untreated) organoids (Figure 5A; Table S3). The heatmap of these DEPs revealed that while most organoids clustered according to treatment status, a subset of treated organoids (NCI‐3, AD‐17, AD‐18, ADNPS‐19, and ADNPS‐20) clustered with the untreated samples (Figure 5B). PCA of these DEPs also confirmed that the proteomics profiles of these samples cluster into distinct groups. EO‐treated organoids from AD patients and AD patients with NPSs (AD+NPS) formed one distinct cluster, and EO‐treated organoids from NCI participants formed another. While untreated organoids mostly clustered together, NCI‐3, AD‐17, AD‐18, ADNPS‐19, and ADNPS‐20 could also be identified within a potentially separate cluster (Figure 5C), suggesting that these samples did not effectively respond to EO exposure.

STRING (Figure 5D) determined that there are no significant interactions or GO enrichments between or among these proteins (STRING enrichment, *p* = 0.0573); the only significant functional enrichment in this network was a UniProt keyword, “phosphoprotein” (FDR = 0.0006; Figure 5D; Table S3). It is not surprising to see enrichment of phosphoproteins in this list of EO‐mediated DEPs, given that EO and other SSRIs influence phosphorylation processes, particularly those related to neuroplasticity and antidepressant effects.^63^, ^64^, ^65^, ^66^, ^67^ However, it is encouraging that our patient‐derived organoids may reflect aspects of relevant SSRI‐mediated biological processes.

### 3.6 Differential protein expression profiles in EVs released by 5‐HT organoids treated with EO reveals patient heterogeneity in drug response

In parallel, we examined the EVs released by the EO‐treated organoids. Because EVs have been proposed as accessible biomarkers for both disease and treatment response, we reasoned that proteomic profiling of EVs could reveal signatures of EO‐induced effects. By assessing whether drug‐related changes are detectable in EVs, we aimed to assess if EVs could serve as minimally invasive indicators of drug effects in future therapeutic screening efforts.

**FIGURE 6:**
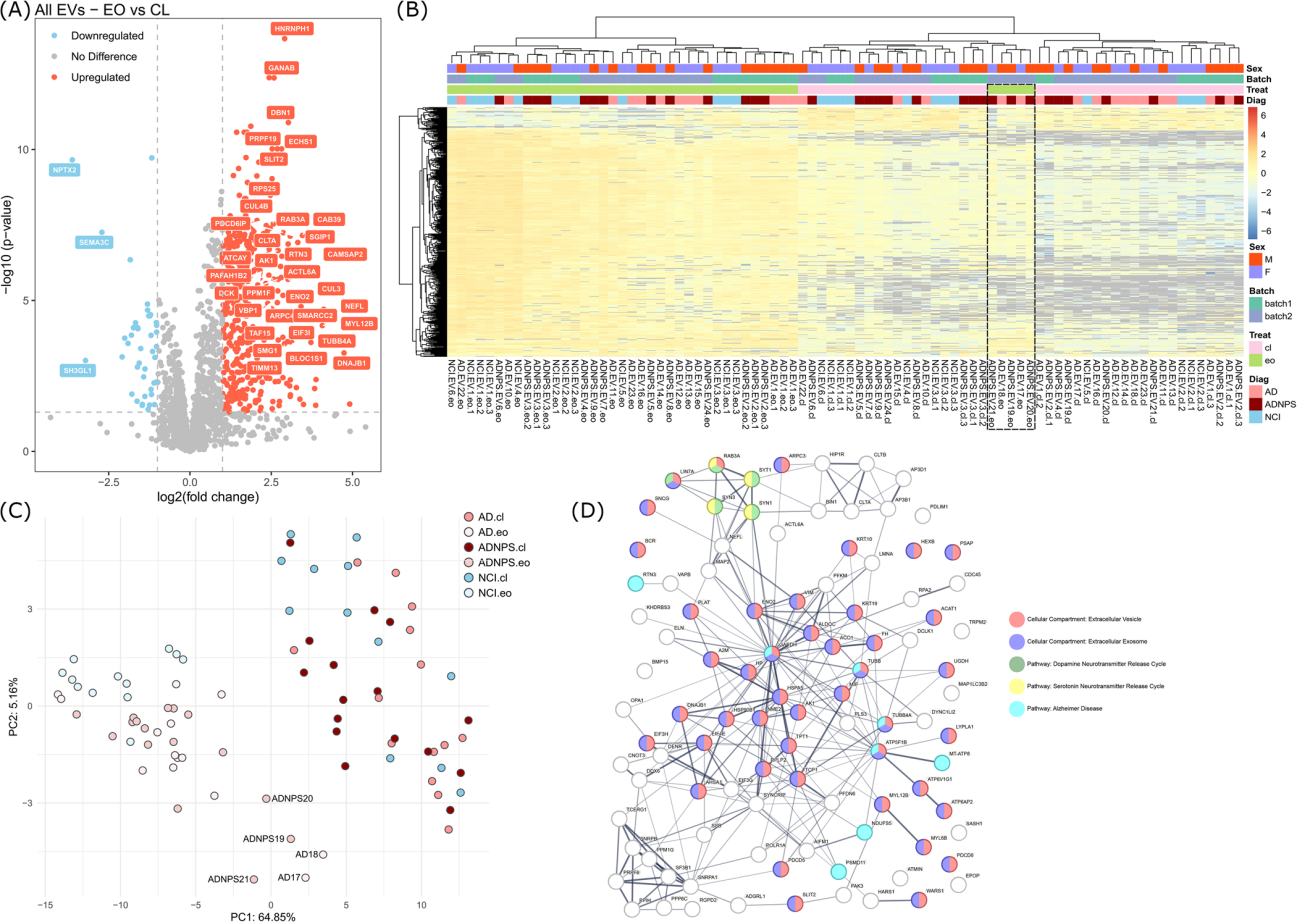
Comparative proteomic profiling of EVs released from EO‐treated 5‐HT organoids and no treatment control organoids. A, A volcano plot showing the log_2_ fold change and ‐log_10_(*P* value) of protein expression in EVs exposed to EO, relative to untreated EVs. Red = DEPs with *P*adj < 0.05 and log_2_FC > 1; Blue = Red = DEPs with *P*adj < 0.05 and log_2_FC < 1, using a paired two‐tailed Student *t* test. Labeled proteins = *P*adj < 0.001 and |log_2_FC| > 2.5. B, A heatmap showing proteomics expression profiles of the 458 DEPs between EO‐treated and untreated EVs. Sample classification structure: Diagnosis.SampleType.BiologicalReplicate.Treatment.TechnicalReplicate (e.g., NCI.EV.1.cl.1 = No Cognitive Impairment, Extracellular Vesicle, Biological Replicate 1, Control, Technical Replicate 1). C, PCA demonstrates that EO‐treated and control EVs cluster independently, but that a subset of EO‐treated EVs may be unresponsive to the drug. D, Using a hypergeometric test with FDR correction, STRING analysis demonstrated many significantly enriched GO terms and pathway enrichments among the top 100 DEPs, including “extracellular vesicle” (pink), “extracellular exosome” (purple), “dopamine neurotransmitter release cycle” (green), “serotonin neurotransmitter release cycle” (yellow), and “Alzheimer disease” (teal). 5‐HT, serotonergic; AD, Alzheimer's disease; cl, control; DEP, differentially expressed protein; Diag, diagnosis; EO, escitalopram oxalate; FDR, false discovery rate; GO, Gene Ontology; NCI, no cognitive impairment; NPS, neuropsychiatric symptoms; PCA, principal component analysis; Treat, treatment.

Using our threshold of *P*adj < 0.05 and |log_2_FoldChange| > 1 on our filtered dataset, we identified 458 DEPs in the EVs from EO‐treated organoids compared to the EVs from untreated organoids. Of these, 415 were significantly upregulated, and 43 were significantly downregulated (Figure 6A; Table S4). Compared to the results we observed in the organoid samples, the heatmap of the DEPs in EVs revealed a very similar pattern of response to EO exposure. Again, while most corresponding EVs clustered according to treatment status, a subset of treated EVs (AD‐17, AD‐18, ADNPS‐19, ADNPS‐20, and ADNPS‐21) clustered more closely with the untreated samples (Figure 6B). PCA of these DEPs also confirmed that the proteomics profiles of these samples cluster into distinct groups. EO‐treated EVs formed one distinct cluster, and untreated EVs clearly formed another; however, just like in the organoids, AD‐17, AD‐18, ADNPS‐19, and ADNPS‐20 formed a third cluster, but this time alongside ADNPS‐21 instead of NCI‐3 (Figure 6C). This suggests that our brain organoids and their corresponding EVs represent models capable of detecting drug response variability in patient subpopulations.

STRING analysis showed that these DEPs are highly interconnected (STRING enrichment, *p* < 1 × 10^−16^), with 1.92‐fold more interconnections than expected by chance for the same number of proteins. Among a long list of 1175 significant functional enrichments in this network of proteins (Table S4), notable enrichments include GO cellular component “extracellular vesicle” (FDR = 7.65 × 10^−28^) and “extracellular exosome” (FDR = 9.84 × 10^−28^); GO molecular function “RNA binding” (FDR = 2.63 × 10^−27^) and “protein binding” (FDR = 1.43 × 10^−16^); GO biological function “RNA splicing” (FDR = 8.06 × 10^−12^) and “regulation of protein metabolic process” (FDR = 6.47 × 10^−11^); KEGG pathways “Alzheimer disease” (FDR = 0.0178); and reactome pathways “dopamine neurotransmitter release cycle” (FDR = 0.0052) and “serotonin neurotransmitter release cycle” (FDR = 0.0147). These results further strengthen our confidence in the cell‐type specificity of our EV separation protocol (“extracellular vesicle”; “extracellular exosome”) and demonstrate that the statistical thresholds applied to our proteomics data effectively capture RNA dysfunction and major biological processes affected by disease status (“Alzheimer disease”) and drug exposure (“dopamine neurotransmitter release cycle”; “serotonin neurotransmitter release cycle”). For ease of viewing such a large network, we generated a STRING network plot using only the top 100 DEPs with *P*adj < 0.01 and |log_2_FoldChange| > 1 (Figure 6D). Together, these results suggest that EO treatment has a substantial impact on EV biology that is variable across individuals, as indicated by the strong enrichment for EV‐related terms among DEPs.

## 4 DISCUSSION

We demonstrate the utility of brain organoids and their corresponding EVs as reliable, physiologically relevant, preclinical models for precision medicine approaches. We show that AD‐derived organoids and EVs, even from 30 individual cell lines, reveal common DEPs relevant to AD pathophysiology, demonstrating that these models are sensitive to detect preclinical changes in disease‐relevant pathways.

Despite using strict statistical thresholds, we detected several notable DEPs that were strongly associated with AD risk. In organoids, we observed 16 DEPs, including significant downregulation of SEMA4C, a member of the class 4 semaphorin family that shares structural similarities and overlapping roles in immune regulation and neural development with SEMA4D.^68^ While SEMA4D is upregulated in neurons during the progression of neurodegenerative diseases such as AD—promoting astrocyte reactivity and neuroinflammation^69^—SEMA4C downregulation may reflect a loss of neuroprotective semaphorin signaling in AD, underscoring the divergent roles of these family members in disease pathogenesis. Notably, a SEMA4D‐blocking antibody, pepinemab (Vaccinex),^70^ has recently shown safety and promising biological and cognitive effects in a Phase 1b/2 clinical trial in early AD (https://clinicaltrials.gov/study/NCT04381468; NCT04381468), further supporting the therapeutic potential of targeting semaphorin pathways in AD.

We also show that brain organoid‐derived EVs are a biologically relevant model by which to identify individuals and patient subtypes who might benefit from targeting specific genes or modes of disease pathogenesis. In EVs, we identified 28 DEPs, including significant downregulation of RAB3A, NSF, ATCAY, and COL5A1, proteins whose corresponding genes have been highlighted in the Agora database as potential AD therapeutic targets. Specifically, RAB3A, NSF, and ATCAY agonism and COL5A1 antagonism have been predicted to reduce AD progression.^48^ COL5A1 is an extracellular matrix associated protein and member of a transcriptional module (M42; “matrisome”) significantly associated with AD neuropathology and cognition.^61^ ATCAY (caytaxin) is a neuron‐specific adaptor protein that associates with kinesin‐1 and is implicated in the intracellular transport of vesicles and organelles, suggesting a role in regulating the trafficking of synaptic components essential for neuronal communication.^71^ NSF is an ATPase involved in fusing vesicles with the plasma membrane,^72^ downregulation of which is hypothesized to impact learning and memory by impairing synaptic function, including disruptions in neurotransmitter release and long‐term potentiation.^73^ Furthermore, tau‐dependent inhibition of NSF has been associated with learning deficits in rodent models of AD.^74^ RAB3A is another synaptic vesicle protein that, alongside APP and presenilin 1, is known to be downregulated in the AD brain,^59^, ^75^ and loss of which is likely involved in disease‐associated synaptic dysfunction. Intriguingly, RAB3A and ATCAY are also among significantly DEPs that were found to be upregulated in response to EO, a known SSRI. In this context, upregulated RAB3A was specifically enriched in pathways involving the serotonin neurotransmitter release cycle, neuronal projections, and synaptic vesicles, and upregulated ATCAY was implicated in pathways involving synaptic vesicles, neurogenesis, and regulation of metabolism and organelles. That these targets emerged from our unbiased proteomic analysis of our organoids and their EVs strongly supports the biological validity of our model system.

Overall, the greater number of disease‐relevant DEPs in EVs indicate that EVs capture a broader spectrum of disease‐associated proteomic changes compared to the organoids from which they are derived. The greater pathway enrichment in EVs suggests that they may act as concentrated reporters of neuronal dysfunction and intercellular signaling, reflecting AD‐associated molecular perturbations that are partially diluted in whole organoids. These findings underscore the potential of organoid‐derived EVs as preclinical models capable of capturing early dysregulation of pathways involved in learning, memory, and neurotransmitter release, as well as the modulation of drug‐targetable pathways, offering a complementary readout to neuronal proteomes.

Our results also demonstrate that brain organoids, and especially their corresponding EVs, are sensitive to detect individual response to EO exposure across our large organoid cohort. Notably, enrichment of pathways related to dopamine and serotonin neurotransmitter release in the EO‐treated samples supports EO's known mechanism of action as an SSRI. Interestingly, despite EO not being designed as an anti‐AD drug, we also observed enrichment in the KEGG AD pathway. Although we cannot yet determine the functional relevance of this finding, it raises intriguing possibilities regarding how EO may intersect with AD‐relevant biology in patient‐derived samples.

Consistent with numerous prior studies highlighting variability in SSRI efficacy,^8^, ^10^, ^31^, ^32^ our organoids, and their corresponding EVs, were unable to detect distinct proteomic signatures indicative of preferential response to EO in the AD+NPS subpopulation, further underscoring the pronounced individual heterogeneity in serotonergic drug response. Therefore, our results underscore the potential of patient‐derived brain organoids and EVs as a preclinical screening platform to identify treatment‐responsive subgroups prior to costly and time‐intensive clinical trials.

In further support of this point, EO elicited heterogeneous molecular responses across both organoid and EV proteomes. While some lines exhibited significant changes in protein expression after EO exposure—particularly in pathways related to synaptic vesicle cycling, serotonin signaling, and transcriptional/translational regulation—a subset of patient‐derived lines (AD‐17, AD‐18, ADNPS‐19, and ADNPS‐20) exhibited minimal proteomic changes that did not significantly differ from controls. This suggests the existence of non‐responder phenotypes at the molecular level, which highlights the variability in response to SSRIs that are used to treat NPSs.

Our study represents the largest brain organoid investigation reported to date, using 5‐HT hindbrain organoids, and corresponding EVs, derived from 30 individual donor iPSC lines. Our cohort captures an unprecedented degree of biologic heterogeneity, which is especially critical in the context of AD, in which clinical heterogeneity is a defining feature. While this scale enables assessment of inter‐individual variability in molecular responses and enhances the translational relevance of brain organoids and their corresponding EVs as preclinical models, advancing precision medicine in AD will require scaling these efforts further. The development of high‐throughput screening methods capable of evaluating larger numbers of patient‐derived organoids and EVs will ultimately support more comprehensive and individualized disease modeling and therapeutic discovery.

Our study is also the first to compare analogous proteomics data from brain organoids and their corresponding EVs. We demonstrate that our 5‐HT hindbrain organoids display robust expression of both neuronal and serotonergic proteins across 30 genetically distinct donor lines, spanning AD patients and controls, validating the reliability of our differentiation protocol and proteomics data. Likewise, we show that EVs derived from these organoids reproducibly express known EV and NDEV markers, confirming their cellular origin and integrity.

Nonetheless, patient‐derived organoid systems have limitations, including lack of vasculature, restricted neuronal circuit complexity, and incomplete cellular maturation, which limits modeling of late‐stage AD phenotypes. Furthermore, the organoids in this study are predominantly neuronal, and we recognize that other cell types, including astrocytes, microglia, and endothelial cells, play important roles in synaptic regulation, neuroinflammation, and vascular function, all of which can modulate pharmacological responses. Incorporating these cell types in more complex 3D multicellular systems, such as assembloids, could provide a more physiologically relevant environment and potentially reveal additional layers of heterogeneity in drug response.

Furthermore, direct correlation with clinical phenotypes remains a significant challenge, as cognitive trajectories, neuropsychiatric outcomes, and biomarker profiles in AD patients are influenced by complex factors, including gene–environment interactions. While our findings demonstrate that organoids and their EVs capture both shared and divergent molecular signatures of disease status or response to pharmacological perturbation, direct comparisons of SSRI therapeutic efficacy with drug‐induced molecular changes in EVs versus matched brain tissue in clinical settings remain limited and may represent valuable avenues for future investigation. The preclinical platform we describe in this study can thus be used to complement other models and inform studies of inter‐individual heterogeneity in AD.

Together, our results highlight the feasibility and value of large‐scale patient‐derived brain organoid models and their corresponding EVs as preclinical models of AD risk that can be used to investigate variability of disease risk and mechanisms, as well as drug effects in the human brain. This work positions patient‐derived organoid‐EV platforms as transformative tools for precision medicine. Their ability to model both shared pathophysiology and individual variability addresses critical gaps in drug discovery, offering a time‐ and cost‐effective strategy to improve clinical trial success rates. As the field continues to seek reliable models for high‐throughput therapeutic screening, our approach enables scalable biomarker discovery, therapeutic validation, and personalized intervention strategies for neurodegenerative diseases.

## AUTHOR CONTRIBUTIONS

Vasiliki Mahairaki and Constantine G. Lyketsos conceptualized the study. Ram Sagar generated and characterized all iPSC lines included in the paper. Cristina Zivko (see Acknowledgments section) performed differentiation of the iPSC lines into 5‐HT organoids and applied drug. Ram Sagar, Cristina Zivko, and Anton Iliuk (on behalf of Tymora Analytical Operations) isolated extracellular vesicles. Anton Iliuk prepared EV and organoid samples for proteomics, processed LC‐MS/MS data, and performed label‐free quantitation analysis. Daiyun Dong performed data processing, batch effect correction, and normalization. Rachel J. Boyd and Daiyun Dong designed and performed bioinformatics pipelines, data analysis, and figure generation. Rachel J. Boyd wrote and assembled the manuscript. Rachel J. Boyd, Ram Sagar, Cristina Zivko, Anton P. Porsteinsson, Paul B. Rosenberg, Constantine G. Lyketsos, Waqar Ahmed, Xenia Androni, and Vasiliki Mahairaki contributed to scientific discussion and edits.

## FUNDING INFORMATION

The study funders had no role in the study design; in the collection, analysis, or interpretation of data; in the writing of the report; or in the decision to submit the paper for publication. Rachel J. Boyd received funding from NIH (T32 AG058527, Translational Aging Research Training Program). Anton P. Porsteinsson received funding from NIH (R01AG052510, S‐CitAD). Paul B. Rosenberg received funding from NIA (AGR01054771, AGR01050515, AGR01046543, and AGR01071522). Constantine G. Lyketsos received funding from NIH (R01AG052510, S‐CitAD; P30AG066507, JHADRC). Kenneth W. Witwer received funding from Paul G. Allen Frontiers Foundation and The Richman Family Precision Medicine Center of Excellence in Alzheimer's Disease at Johns Hopkins University. Vasiliki Mahairaki received funding from NIH (1RF1AG083801) and The Richman Family Precision Medicine Center of Excellence in Alzheimer's Disease at Johns Hopkins University.

## CONFLICT OF INTEREST STATEMENT

Dr. Lyketsos has served or serves as consultant or advisor to Roche, Avanir, Karuna, Maplight, Axsome, GW Research Limited, Merck, EXCIVA GmbH, Otsuka, IntraCellular Therapies, Medesis, BMS, and IQVIA. Dr. Rosenberg has received research grants from the National Institute on Aging, Alzheimer's Clinical Trials Consortium, Richman Family Precision Medicine Center of Excellence on Alzheimer's Disease, Eisai, Functional Neuromodulation, and Lilly; honoraria from Lilly, GLG, Leerink, Medalink, Novo Nordisk, Noble Insights, TwoLabs, Otsuka, MedaCorp, ExpertConnect, HMP Global, Worldwide Clinical Trials, Medscape, and Neurology Week. Dr. Porsteinsson reports personal fees from Acadia Pharmaceuticals, Athira, Axsome, Biogen, BMS, Cognitive Research Corp, Cognition Therapeutics, Eisai, IQVIA, Lundbeck, MapLight Therapeutics, Novartis, Novo Nordisk, Oligomerix, ONO Pharmaceuticals, Otsuka, WCG, WebMD, and Xenon; and grants to his institution from Alector, Athira, Biogen, Cassava, Eisai, Eli Lilly, Genentech/Roche, Vaccinex, NIA, NIMH, and DOD. Dr. Witwer is president of the International Society for Extracellular Vesicles; is or has been an advisory board member of B4 RNA, Everly Bio, Interactome Biotherapeutics, NeuroDex, and NovaDip; and holds stock options with NeuroDex. Anton Iliuk is the founder, president, and CTO of Tymora Analytical Operations. Rachel J. Boyd, Daiyun Dong, Ram Sagar, Waqar Ahmed, Xenia Androni, and Vasiliki Mahairaki have nothing to disclose. Author disclosures are available in the supporting information.

## CONSENT STATEMENT

Induced human iPSCs used in this project have been generated from peripheral blood monocytes obtained upon fully informed consent from human subjects as outlined by the JHMI eIRB‐approved research study application (NA_00045104) and consent process. All experiments were conducted according to regulations governing the use of human stem cell lines in biomedical research.

## Supporting information



Supporting information

Supporting information

Supporting information

## Data Availability

The R scripts used for the analyses and visualizations is available at: https://github.com/daiyundong/Machairaki_2025_EV_Organoid_Proteomics The website based on Shiny that allows readers to explore our data is available at: https://jtbuuy‐daiyun‐dong.shinyapps.io/organoid_proteomics_batch_correction_shiny/
