# Supplementary material for: Proteomic profiling of brain organoids and extracellular vesicles identifies early Alzheimer's disease biomarkers and drug response heterogeneity
Source: Alzheimers Dement. 2026 Apr 8;22(4):e71273. doi: 10.1002/alz.71273 (PMC13058922; doi:10.1002/alz.71273)
Supplement: Supplementary file 2 — Supporting information [file ALZ-22-e71273-s001.docx]

**Supplementary Materials**


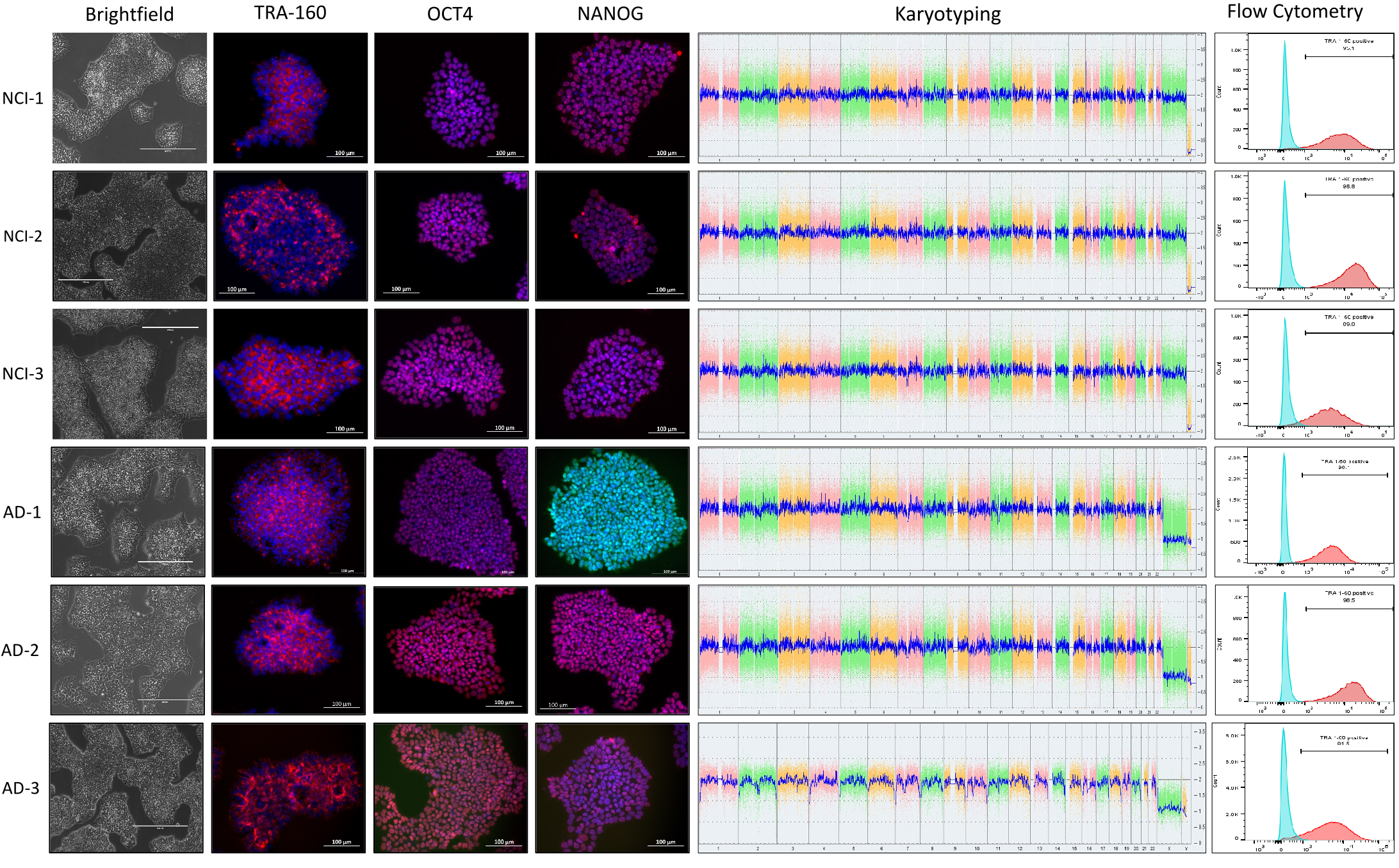


**Supplementary Fig. 1.** Representative characterization of n = 3 iPSC lines derived from patients without cognitive impairment (NCI-1, NCI-2, NCI-3) and n = 3 iPSC lines derived from patients diagnosed with Alzheimer’s disease (AD-1, AD-2, AD-3) using immunocytochemistry staining for TRA-160, OCT4, and NANOG, karyotyping, and flow cytometry. These results have been reported in previously published manuscripts where we have utilised the same iPSC lines^9,35^.


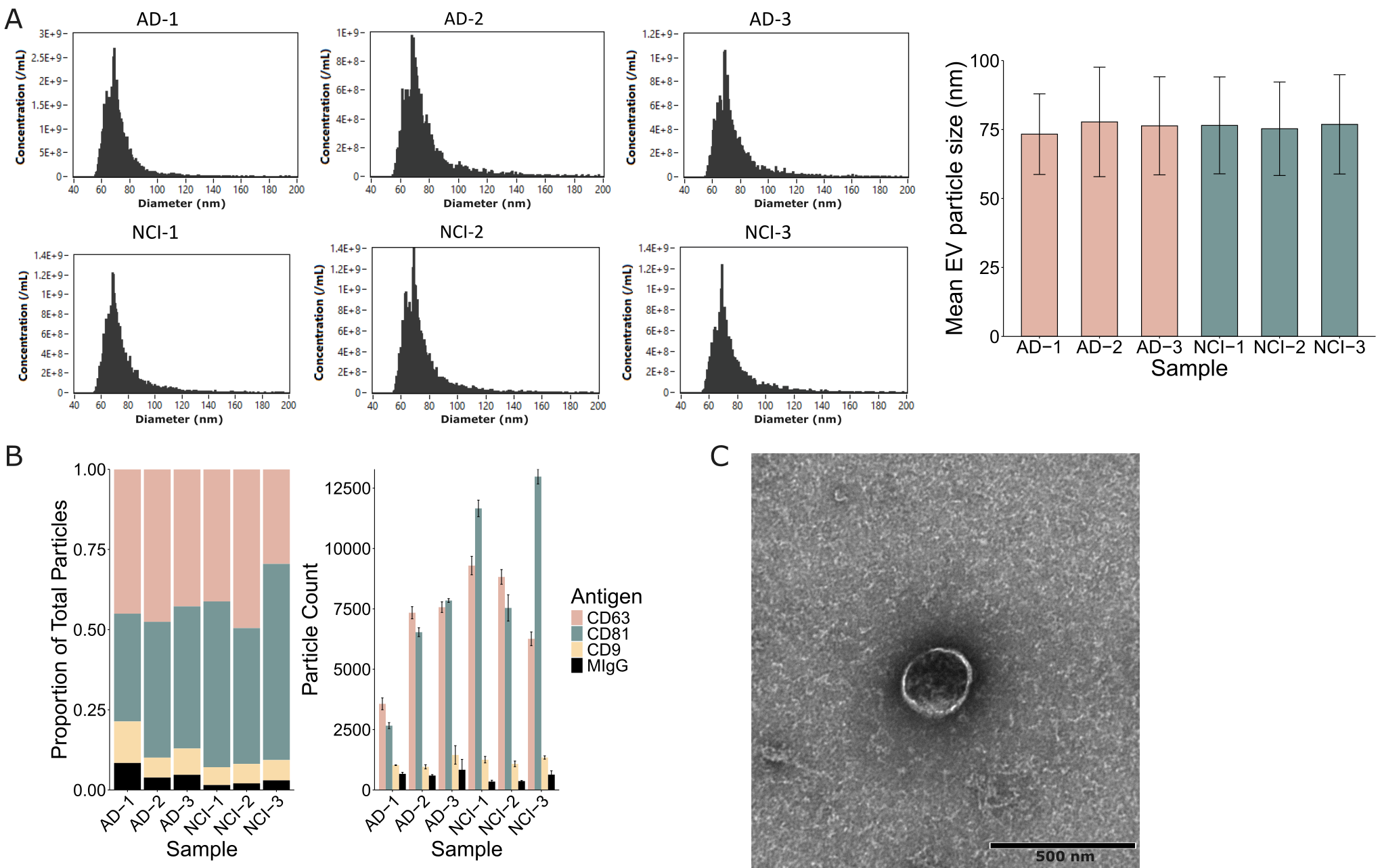


**Supplementary Fig. 2.** (**A**) Characterization of particle diameter (nm) distribution within EV preparations separated from n = 3 hindbrain organoids derived from patients without cognitive impairment (NCI-1, NCI-2, NCI-3) and n = 3 hindbrain organoids derived from patients diagnosed with Alzheimer’s disease (AD-1, AD-2, AD-3) by NanoFCM. (**B**) Interferometry reveals the number of EVs captured by antibodies to tetraspanins CD63, CD81, and CD9, or by a non-specific control antibody (MIgG) as assessed by Single-Particle Interferometric Reflectance Imaging Sensor (SP-IRIS). **(C)** Representative negative staining by transmission electron microscopy (TEM) to visualize EV particles.
